# New Recessive Syndrome of Microcephaly, Cerebellar Hypoplasia, and Congenital Heart Conduction Defect

**DOI:** 10.1002/ajmg.a.34078

**Published:** 2011-10-14

**Authors:** Maha S Zaki, Ghada M H Abdel Salam, Sahar N Saleem, William B Dobyns, Mahmoud Y Issa, Shifteh Sattar, Joseph G Gleeson

**Affiliations:** 1 Clinical Genetics Department, Human Genetics and Genome Research Division, National Research CentreCairo, Egypt; 2 Radiology Department, Cairo UniversityCairo, Egypt; 3 Center for Integrative Brain Research, Seattle Children's Research Institute, Seattle Children's Hospital, University of WashingtonSeattle, Washington; 4 Neurogenetics Laboratory, Department of Neurosciences, Howard Hughes Medical Institute, University of CaliforniaySan Diego, California

**Keywords:** microcephaly, insulin-dependent diabetes, cerebellar hypoplasia, mental retardation, heart block

## Abstract

We identified a two-branch consanguineous family in which four affected members (three females and one male) presented with constitutive growth delay, severe psychomotor retardation, microcephaly, cerebellar hypoplasia, and second-degree heart block. They also shared distinct facial features and similar appearance of their hands and feet. Childhood-onset insulin-dependent diabetes mellitus developed in one affected child around the age of 9 years. Molecular analysis excluded mutations in potentially related genes such as *PTF1A*, *EIF2AK3*, *EOMES*, and *WDR62*. This condition appears to be unique of other known conditions, suggesting a unique clinical entity of autosomal recessive mode of inheritance. © 2011 Wiley Periodicals, Inc.

## INTRODUCTION

Recessive microcephaly (i.e., reduced brain volume) can be seen in isolation (primary microcephaly) or in association with cortical migration defects (microlissencephaly), with dwarfism (microcephalic osteodysplastic primordial dwarfism type Majeweski II) or with brainstem and/or cerebellar malformations [Barkovich et al., 1998; Kaindl et al., 2010]. Brain MRI in microcephaly is frequently diagnostic of a particular genetic entity, and may show simplification of the gyral pattern, as well as thin corpus callosum. Developmental delay of cognition and motor systems are routinely observed when the OFC is below the third centile and epilepsy and spasticity may be observed in association with migration defects or brainstem disorders [Basel-Vanagaite and Dobyns, 2010].

Second-degree heart block is diagnosable if one or more (but not all) atrial impulses fail to be transmitted to the ventricles due to impaired conduction. There are two distinct types of second-degree AV block, called Mobitz I (Wenckebach) and Mobitz type II. In both types, a P-wave is partially blocked from forming a QRS complex, but in Mobitz I there are increasing PR interval delays in each cardiac cycle until omission of a QRS complex; it is due to AV nodal defects. In Mobitz II there is no such pattern, and it is caused by His-Purkinje system defects [Keane et al., 2006]. Type I is more commonly associated with bradycardia, whereas type II is more likely to progress to complete heart block, where the escape rhythm fails.

Here we report on a consanguineous family in which microcephaly with cerebellar hypoplasia was seen together with cardiac conduction on defects in four affected children who also had specific facial features, fusiform fingers and overlapping toes, findings not observed in the healthy siblings.

## METHODS

DNA was isolated from peripheral blood using a Qiagen Genomic DNA extraction kit. PCR and sequencing was performed using standard methods. SNP markers were identified using the current version of the Human Genome Browser (http://www.genome.ucsc.edu) as the closest to the gene of interest from the standard mapping set defined by the HapMap project [Murray et al., 2004]. For each gene to be excluded, we genotyped a set of SNP markers within 5 cM of each relevant gene, and identified the closest polymorphic markers. We utilized the Haldane mapping function to exclude potential genes, by converting theta values giving a negative linkage test into genetic distances to exclude regions on either side of a polymorphic marker [Terwilliger and Ott, 1994]. For all candidate genes examined, zLOD scores were below −2, thus excluding linkage to loci containing these candidate genes.

## CLINICAL REPORTS

### Family History

**FIG. 1:**
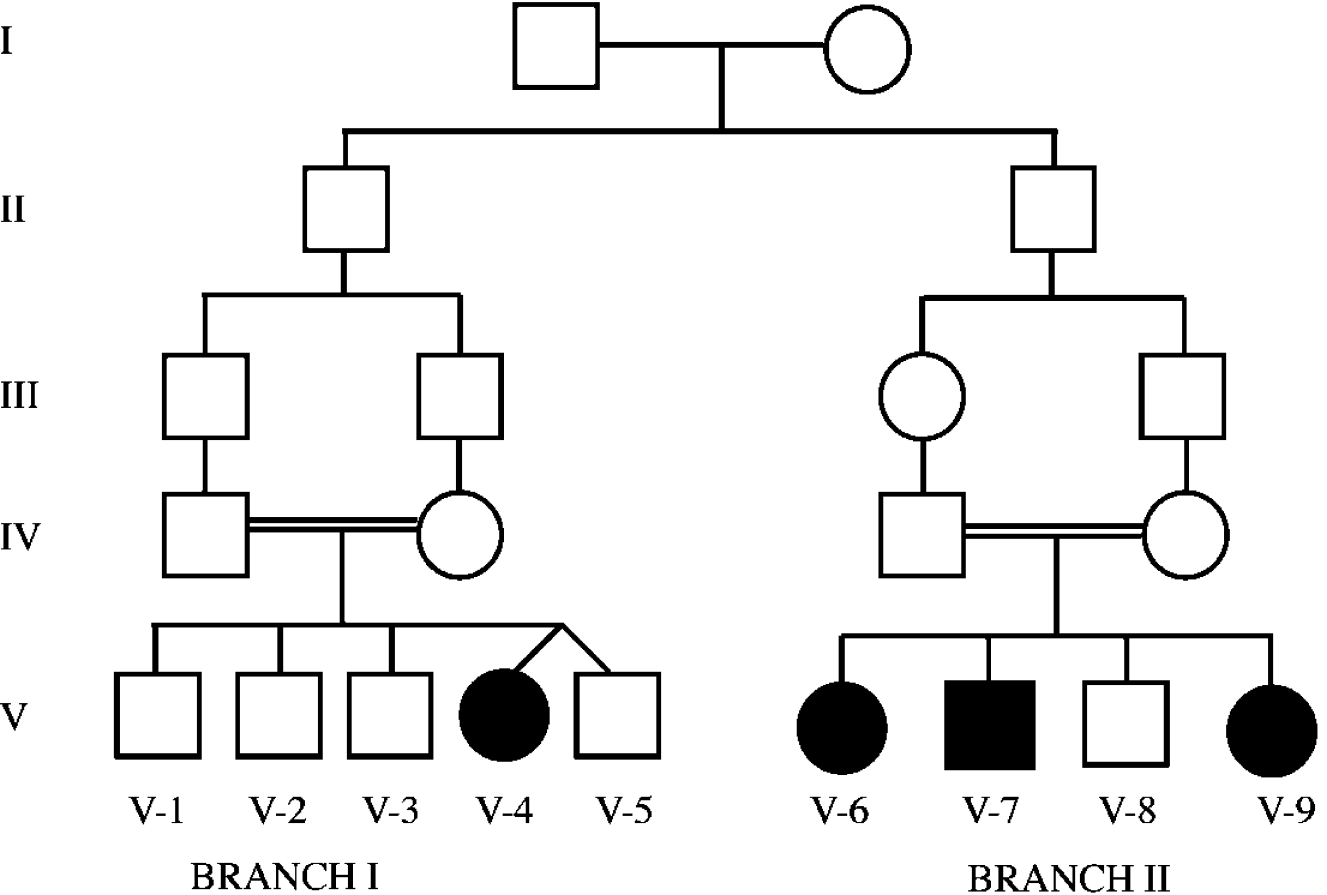
Pedigree of the family. Two first-cousin marriages produced two branches (Branch I and Branch II) each with affected members from a common founder.

Family CBH-348 is of Upper Egyptian origin (Fig. 1). They presented to Neurogenetics Clinic with three affected children (V-4, -6, -7) manifesting growth failure, developmental delay, and severe cognitive impairment. Subsequently there was birth of an additional affected girl (V-9) (Table I). No history of abortions or deaths was recorded. The parents of both affected branches were healthy with normal appearance, cognition, cardiac, and endocrine function. The paternal and maternal ages for Branch I were 43 and 38 years old, while for Branch II were 40 and 33 years old, respectively. Their anthropometric measurements revealed average height, weight, and head circumference. For Branch I, maternal height was 165 cm (+0.5 SD), weight 61 kg (+0.4 SD), head circumference 54 cm (mean) and parental height was 175 cm (mean), weight 78 kg (+1.5 SD), head circumference 56 cm (+0.7 SD). For Branch II, maternal height was 156 cm (−1 SD), weight 56 kg (mean), head circumference 54 cm (mean) and paternal height was 170 cm (−0.7 SD), weight 72 kg (+1 SD), and head circumference 55 cm (mean). Electrocardiogram (ECG) for parents and non-affected sibs were normal, excluding heart conductivity defects.

**TABLE I:** Main Findings Among the Studied Patients With This Unique Autosomal Recessive Syndrome

Findings	Patient V-4	Patient V-6	Patient V-7	Patient V-9
Sex	Female	Female	Male	Female
Age	8 1/12y	14y	11y	7 month
Weight (SD)	19.9 kg (−3)	29 kg (−3.5)	21 kg (−3)	5 kg (−2.9)
Length (SD)	96 cm (−5)	115 cm (−7.5)	107 cm (−5.8)	58 cm (−3.9)
OFC (SD)	36.8 cm (−11)	40 cm (−11.5)	38.5 cm (−10)	32 cm (−8.2)
Mental retardation	Profound	Profound	Profound	Profound
Autistic-like behavior	+	+	+	−
Specific facial features	+	+	+	+
Long tapered fingers	+	+	+	+
Camptodactyly	+	−	+	−
Crowded toes	+	+	+	+
Vasomotor instability	+	+	+	+
Bradychardia	+	+	+	−
ECG: 2nd degree heart block	+/Mobitz I	+	+	−
Neurological: Hypotonia	+	+	+	+
Brisk reflexes	+	+	+	+
Dystonic like movements	Prominent	Mild	Moderate	Mild
Cerebellar manifestations	+	+	+	+
CBHA	+	+	+	+
Simplified gyral pattern	+	+	+	+
Thin Corpus callosum	+	+	+	+
HMSA	+	+	+	+
Dysplastic occipital lobe	−	+	−	+
Karyotyping	46, XX	46, XX	46, XY	46, XX
Metabolic screening	WNL	WNL	WNL	WNL
GTT	N/A (IDDM)	High normal	High normal	Random normal

CBHA, cerebellar hypoplasia/atrophy; GTT, glucose tolerance test; IDDM, insulin-dependent diabetes mellitus; N/A, not available; OFC, occipital-frontal head circumference; WMSA, white matter signal abnormalities; WNL, within normal limits.

### Patient 1 (V-4)

**FIG. 2:**
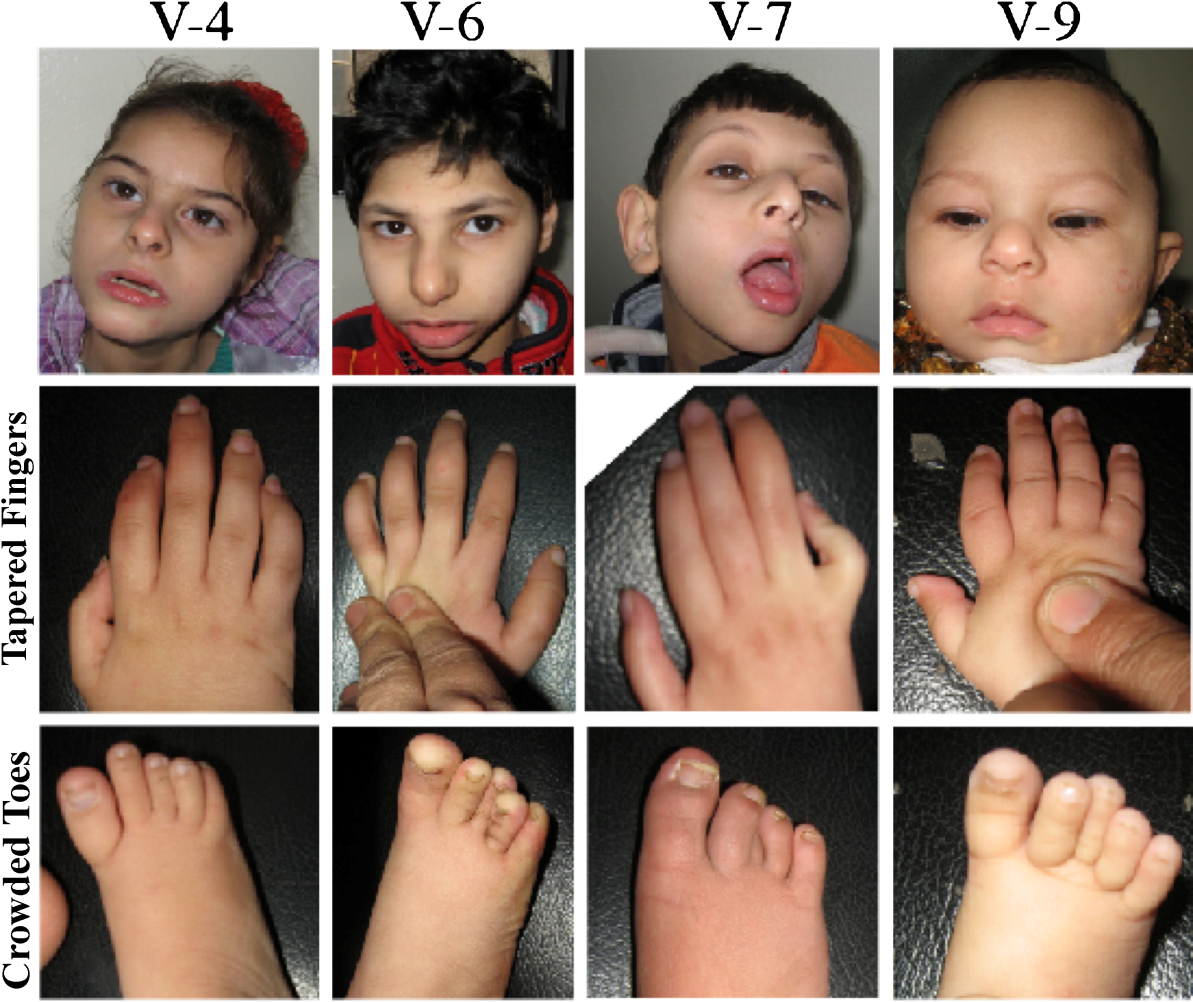
Unique syndromic features of four affecteds from family CBH-348. Top: Wide forehead, prominent nose with prominent collumella, V-shaped thin upper lip, prominent lower lips, long philtrum, and large ears were noted in all affecteds. Middle: Hands showed fusiform long fingers in all affecteds, campto-clinodactyly of fifth finger in V-4 and V-7 and clinodatyly of fifth finger in V-6. Bottom: Crowded toes.

Patient 1 was an 8 1/12-year-old female (Fig. 2); part of a non-identical twin and a product of full term uneventful gestation. Her small head was noted at birth with a head circumference of 26.7 cm (−4.5 SD) while weight and height were 2.2 kg (−2.1 SD) and 45.5 cm (−2.1 SD), respectively. All development milestones were profoundly delayed. No history of seizures was recorded but she had recurrent attacks of fainting associated with cyanosis and extreme bradychardia that required resuscitation and admission to the ICU. At the time of examination, the patient had growth failure and microcephaly; weight of 19.9 kg (−3 SD), height of 96 cm (−5 SD) and head circumference of 36.8 cm (−11 SD). She was able to sit supported, could not follow or grasp objects, failed to develop language skills and had no sphincteric or bowel control. Autistic behavior and dystonic-like movements of the head and limbs were evident.

The patient had specific facial features including broad forehead, thin long eyebrows, upslanting palpebral fissures, prominent nose, long philtrum, thin vermillion of the upper lip, prominent lower lip, and large ears with prominent antihelix. Hands and feet showed long fusiform fingers with bilateral campto-clinodactly of fifth fingers and crowded toes. General examination revealed bradychardia with normal cardiac rhythm (48 bp) and obvious vasomotor instability with prolonged capillary refill, skin mottling, and acrocyanosis. Neurological examination showed hypotonia, brisk reflexes, and intact sensation. In addition to the frequent dystonic movements, there were cerebellar signs manifested with intention tremors and truncal ataxia.

Investigations included normal chromosomal analysis, extended metabolic screening, fundus examination, visual and brain stem auditory evoked potentials, EEG, nerve conduction velocity, and electromyography. Normal creatine kinase, liver and kidney function tests, and thyroid profile were also recorded. Intellectual quotient (IQ) using Stanford–Binet test showed profound mental retardation (score 15). Assessment of cardiac function and anatomy was normal by echocardiography while ECG verified a persistent but intermittent 2:1 AV heart block confirmed by Holter examination. Repeat ECG uncovered a Mobitz I heart block, with successive prolongation of the PR interval (Fig. 4).

**FIG. 3:**
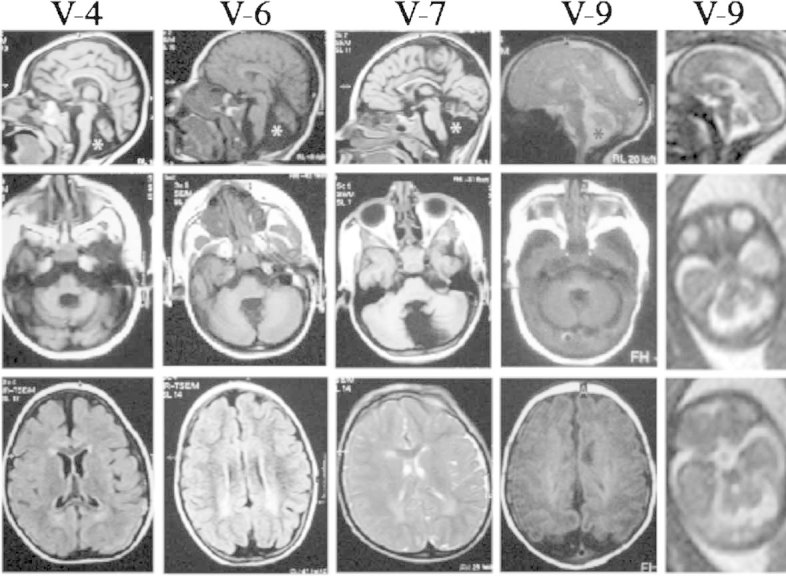
Brain MRI for present patients: First column, patient V-4; second column V-6; third column V-8; fourth column V-9. Top: Brain midline sagittal MR images showing cerebellar hypoplasia/atrophy (arrows) with enlargement of the fourth ventricle (*), thin corpus callosum, and simplified gyral pattern. Middle axial views at the level of the medulla showing failure of the hemispheres to fuse at the midline (arrowheads). Bottom: Axial views at the level of cerebrum, showing simplified gyral pattern and high signal of deep white matter indicating defective myelination. Dysplastic occipital lobe bilaterally is present in both V-6 and 9. Fetal MRI for V-9 (far right) taken at 25 weeks, showing cerebellar hypoplasia on sagittal and axial views.

Brain MRI (Fig. 3) showed cerebellum hypoplasia without severe brain stem involvement. The vermis was particularly affected with prominent cerebellar folia suggestive of mild atrophic changes. Simplified gyral pattern and thin corpus callosum were noted. High signal intensities were seen bilaterally in the deep white matter on T2-weighted and FLAIR sequences impressive of defective myelination.

Upon follow-up at the age of 8 8/12, we learned that this patient became severely dehydrated and was subsequently diagnosed with type I insulin-dependent diabetes mellitus.

### Patient 2 (V-6)

A female patient, 14 years old (Fig. 2), was the first child in this family, born by normal spontaneous vaginal delivery after a full-term pregnancy. At birth, weight was 2.5 kg (−1.57 SD), length was 46 cm (−1.8 SD), and head circumference was 27.8 cm (−3.8 SD). Neonatal history was uneventful while infancy and childhood histories showed failure to thrive, notable small head, and severe developmental delay. No history of seizures or fainting attacks was recorded. On examination, the patient could sit alone, respond to sounds, and follow objects but was unable to grasp objects, feed herself, speak or attain sphincteric or bowel control. Physically, her weight was 29 kg (−3.5 SD), height 115 cm (−7.5 SD), head circumference 40 cm (−11.5 SD), and there were no signs of puberty (B1, P1, A1). Facial features showed upslanting palpebral fissures, prominent nose and columella, long philtrum, V-shaped long upper lip, prominent lower lip, and large ears with prominent antihelix. Long tapered fingers, bilateral clinodactyly of fifth fingers, and mal-alignment of toes were noted. Vasomotor instability was obvious especially in lower limbs. General examination was irrelevant except for bradycardia (64 bp) with regular irregularities every three beats. Neurological examination revealed hypotonia, brisk reflexes, normal sensation, and ataxic movements. Dystonic movements of upper and lower limbs were infrequently noted.

**FIG. 4:**
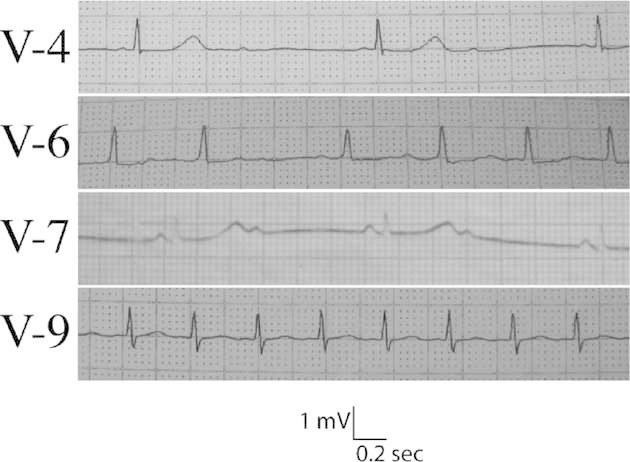
V5 Cardiac traces from affecteds showing heart block in V-4, -6, and -7. V-4 shows Mobitz I heart block, with prolongation of the PR interval followed by a dropped QRS complex. V-6 and -7 show stereotypical pattern of 2:1 heart block and bradycardia. The 2:1 heart block is evident by the appearance of every other P-wave failing to generate a QRS complex, resulting in heart rate of 40–50 beats per minute. V-9 shows no significant abnormalities at this age.

Similar testing to patient V-4 was conducted and results were normal. ECG showed second-degree heart blockage (Fig. 4), IQ score was 20 (profound mental retardation) using Stanford–Binet test, hormonal profile including LH, FSH, estrogen, and progesterone were prepubertal, and glucose tolerance test showed high normal limit. Brain MRI (Fig. 3) revealed similar forebrain and hindbrain as Patient V-4 in the form cerebellar hypoplasia/atrophy with prominent vermian involvement, similar corpus callosum, gyral pattern, and white matter.

### Patient 3 (V-7)

An 11-year-old male patient (Fig. 2), second affected child in branch II, delivered vaginally after uneventful full term pregnancy. Small head (OFC: 28 cm (−4.2 SD)) was noted at birth while weight and height were 2.4 kg (−2.07 SD) and 46.2 cm (−2 SD), respectively. No history of epilepsy, cyanosis, or fainting attacks was recorded. Similarly, patient had severe development delay, he was able to sit alone, had autistic behavior, unable to follow or grasp objects and no language skills acquired. Physical examination reported his weight of 21 kg (−3 SD), height of 107 cm (−5.8 SD), and head circumference of 38.5 cm (−10 SD). Facial features were similar to his affected sib (V-6) showing thin long eyebrows, upslanting palpebral fissures, prominent nose and columella, long philtrum, V-shaped thin vermillion of the upper lip, prominent lower lip, large ears with prominent antihelix. Long fusiform fingers, bilateral fifth finger campto-clinodactyly, crowded toes, and vasomotor instability were detected. The heart exam was normal except for bradycardia (55 bp) with normal cardiac rhythm. Genitalia was normal with prepubertal size testes and no signs of puberty. Neurologically, there were hypotonia, brisk reflexes, frequent dystonic movements of limbs, and truncal ataxia. All investigations were insignificant except that IQ evaluation using Stanford–Binet test was 15 (profound mental retardation), ECG showed second-degree heart block (Fig. 4), and glucose tolerance test showed higher normal limit. Brain MRI was nearly identical forebrain and hindbrain structures as patient V-4 and V-6 (Fig. 3).

### Patient 4 (V-9)

Subsequently, the mother in branch II of the family became pregnant. Because of the presence of three affecteds in the family, serial follow up during the first and second trimesters of pregnancy using ultrasound was performed and examinations were inconclusive. Prenatal MRI was performed at 25th week of gestation with a 1.5 T superconducting system. Images were obtained in three orthogonal planes of the brain and fetal body using ultrafast MRI sequences (Steady State Free Precession and fast spin-echo) to produce T_2_-weighted images with 4 mm thickness and 1 mm overlapping slices. The fetal MR images showed suspicious findings in the form of a small sized skull (at the fifth centile for gestational age), small sized cerebellum (at the fifth centile for gestational age) with its transverse diameter at the fifth centile of normal at the same gestational age as well as suspicious inferior vermis defect. The subarachnoid CSF space overlying the occipital–posterior parietal region was prominent. The corpus callosum appeared thinner than normal. No other fetal abnormalities were detected (Fig. 3). Parents preferred not to terminate pregnancy because of religious and ethical reasons. The child was born vaginally and birth weight was 2.6 kg (−1.5 SD), length was 46 cm (−1.8 SD), and head circumference was 27 cm (−4.3 SD). Patient was evaluated at the age of 3 and 7 months; she showed growth failure and delayed development. At 7 months, her length was 58 cm (−3.9 SD), weight was 5 kg (−2.9 SD), and OFC was 32 cm (−8.2 SD). This patient was very similar to her sibs in appearance, hands and feet, weakness and abnormal movements, however, heart rate was still normal for age with normal ECG pattern (Fig. 4). Postnatal MRI was done at 4 days old (Fig. 3). It revealed cerebellar hypoplasia, simplified gyral pattern, and thin corpus callosum, similar to affecteds in the family. Glucose tolerance test could not be attempted due to the patient's age, but random blood sugar was normal.

### Molecular Analysis

Due to the overlap with Permanent Neonatal Diabetes with Cerebellar Agenesis [OMIM#609069], we excluded the *PTF1A* locus [Sellick et al., 2004] using exclusion mapping with markers rs8341 and rs11813505, both of which were recombinant in this family. Due to the recent report of microcephaly and early onset insulin-dependent diabetes mellitus associated with the *EIF2AK3* gene [de Wit et al., 2006], we analyzed markers rs4832163 and rs735738, both of which were recombinant in the family, which excluded the locus as causative in this family. We similarly excluded the genes *EOMES* and *WDR62*, linked to pleiotropic recessive microcephaly with other cortical and subcortical dysplasias [Baala et al., 2007; Bilguvar et al., 2010; Nicholas et al., 2010; Yu et al., 2010] using neighboring polymorphic markers.

## DISCUSSION

Microcephaly and cerebellar hypoplasia without severe brainstem involvement is caused by recessive mutations in the *EOMES*, *SLC25A19*, and *WDR62* genes. Mutations in *EOMES* (previously known as *TBR2*) give rise to primary microcephaly with corpus callosum agenesis, extensive bilateral polymicrogyria, dilation of the cerebral ventricles, and small cerebellum (OMIM 604615) [Baala et al., 2007]. Mutations in *SLC25A19* give rise to extreme microcephaly, simplified gyral pattern, moderate cerebellar vermis hypoplasia, and 2-ketoglutaric aciduria, reported exclusively as “Amish lethal microcephaly” [Rosenberg et al., 2002]. Recent reports of mutations in *WDR62* as cause of primary microcephaly locus 2 (MCPH2) may be associated with cerebellar involvement [Bilguvar et al., 2010; Nicholas et al., 2010; Yu et al., 2010]. However, the degree of cerebellar hypoplasia and the finding of heart block, makes these conditions unlikely in our patients, and genes were excluded using linkage.

Cerebellar hypoplasia occurs in over 100 syndromes, commonly in Joubert syndrome, defects in protein glycosylation, or in hindbrain patterning. Some of the known causes for cerebellar hypoplasia are related to the “ciliopathies” with frequent co-occurrence of retinal blindness and nephronophthisis, or may be related to congenital disorders of glycosylation. The Dandy–Walker malformation is characterized by enlargement of the fourth ventricle, which was observed in this family, but elevation of the torculum was not observed, arguing against this diagnosis [Barkovich et al., 2009]. The nearly identical imaging findings suggest the same genetic lesion, although they do not suggest a particular genetic pathway. Nevertheless, fetal MR imaging is now acknowledged as the method of choice to delineate posterior fossa malformations in-utero [Saleem and Zaki, 2010], which suggested affection of one of our patients (V-9) while in utero through detection of reduced skull size, reduced transverse cerebellar diameter, and thin corpus callosum. Based on religious and ethical issues, parents preferred not to terminate pregnancy in Patient 4 (V-9); however, suspicious fetal MRI findings enabled family preparations for the birth of another affected child.

The cardiac conduction defect in these patients is consistent with second-degree heart block, and at least for V-4, we can be specific about a Mobitz I subtype. In V-6 and -7, an invariant 2:1 AV block prevented differentiation between Mobitz I and II, but since Mobitz I was observed in V-4, it is most likely the cause in the other two affecteds. Since the older children did not display symptomatic bradycardia until several years of age, and the youngest affected (V-9) has a normal heart rate, it appears that this is an acquired AV block, and that V-9 should be followed closely for this problem. Second-degree block can be observed in several genetic conditions, including microcephaly in Kearns–Sayre syndrome (OMIM#530000) [Ross et al., 1969], but the absence of ophthalmoplegia or biochemical evidence of impaired mitochondrial function argues against this diagnosis. Various forms of progressive familial heart block (PFHB), progressive cardiac conduction defects (PCCD), and atrial septal defect with atrioventricular conduction defects have been described in families with dominant inheritance, implicating the genes *SCN5A*, *NKX2.5*, and *TRPM4* [Benson et al., 1999; Kruse et al., 2009; Schott et al., 1999], but a recessive modes of inheritance has not been reported. The autosomal dominant Holt–Oram syndrome (OMIM#142900) affects the heart and upper limbs and can include conduction defects, but again the clinical patterns are highly distinct.

Diabetes combined with neurological disease is not common, but can be due to mutations in the *PTF1* gene, which causes pancreatic and cerebellar agenesis [Sellick et al., 2004]. Wolcott–Rallison syndrome (OMIM#226980) of multiple epiphyseal dysplasia and early-onset diabetes due to mutations in the *EIF2AK3* gene [Delepine et al., 2000], was recently linked to microcephaly [de Wit et al., 2006], but heart disease is not reported. Further, both of these genes were excluded as candidates based upon SNP mapping studies. Thus, to the best of our knowledge, we suggest that our patients have a unique genetic entity of microcephaly, cerebellar hypoplasia, and heart block inherited in an autosomal recessive fashion.
